# Evaluation of the Protoscolicidal Activity of *Humulus lupulus* Methanolic Extracts on *Echinococcus granulosus Sensu Stricto*

**DOI:** 10.1155/2024/6251666

**Published:** 2024-04-04

**Authors:** Clara María Albani, Azucena Iglesias, Adriana Albanese, Giselle Fuentes, Dalila Orallo, Matías Maggi, María Celina Elissondo

**Affiliations:** ^1^Instituto de Investigaciones en Producción Sanidad y Ambiente (IIPROSAM CONICET-UNMdP), Facultad de Ciencias Exactas y Naturales-UNMdP, Centro Científico Tecnológico Mar del Plata-CONICET, Centro de Asociación Simple CIC PBA, Mar del Plata, Argentina; ^2^Laboratorio de Zoonosis Parasitarias, Facultad de Ciencias Exactas y Naturales (FCEyN), Universidad Nacional de Mar del Plata (UNMdP), Mar del Plata, Buenos Aires, Argentina; ^3^Centro de Investigación en Abejas Sociales, FCEyN, UNMdP, Mar del Plata, Buenos Aires, Argentina; ^4^Departamento de Química y Bioquímica, Facultad de Ciencias Exactas y Naturales, Universidad Nacional de Mar del Plata, Funes 3350, Mar del Plata, Buenos Aires 7600, Argentina

## Abstract

The larval stage of the parasite *Echinococcus granulosus sensu lato* (s.l) is responsible for cystic echinococcosis (CE), a long-term infection affecting humans and animals worldwide, and constitutes a serious public health concern. If left untreated, CE can cause serious damage to multiple organs, especially the liver and lungs. Regarding the treatment, in the last few years, the use of pharmacological treatment has increased, suggesting that in the future, drug therapy may replace surgery for uncomplicated cysts. However, the only available anthelmintic drug to treat this infection is the albendazole, which has an efficacy that does not exceed 50%. On the basis of the above-mentioned evidence, new and improved alternative treatments are urgently needed. The use of natural products and their active fractions and components holds great promise as a valuable resource for the development of novel and effective therapies. Hop (*Humulus lupulus* L.) is a bittering agent in the brewing industry for which the sedative, digestive, anti-inflammatory, and antimicrobial effects have been reported. The purpose of this study was to assess the *in vitro* efficacy of methanolic extracts from the leaves of hop varieties against *E. granulosus sensu stricto* (s.s) protoscoleces. Varieties Mapuche and Victoria caused a stronger protoscolicidal effect compared to the Bullion, Cascade, and Traful varieties (*P* < 0.01), coinciding with their highest content of flavonoids, total polyphenols, and saponins. The viability of protoscoleces treated with the varieties Mapuche and Victoria decreased to approximately 50% at days 5 y 8, respectively, showing alterations such as soma contraction and impaired microtriches. After 18 days of treatment with both varieties, protoscoleces were completely altered both structurally and ultrastructurally. In conclusion, the methanolic extracts of the *H. lupulus* varieties Mapuche and Victoria demonstrated a marked *in vitro* effect against *E. granulosus* s.s. protoscoleces. The beer-making industry exclusively uses hop cones, leaving behind large amounts of hop leaves as an agricultural by-product that is not being utilized. On the basis of our study, we propose that hop leaves could also be used as a source of secondary metabolites with anthelmintic activity.

## 1. Introduction

The larval stage of the parasite *Echinococcus granulosus sensu lato* (s.l) is responsible for cystic echinococcosis (CE), a long-term infection affecting humans and animals worldwide, and constitutes a serious public health concern [1].

The life cycle of *E. granulosus* s.l. is complex and involves an intermediate host (wild or livestock mammals and occasionally humans) and a definitive host (usually dogs and other canids) which contain the adult worm in the intestine and release the eggs along with the feces. Eggs may be accidentally ingested by an intermediary host and develop a hydatid cyst filled with hydatid fluid and protoscoleces [2].

This parasite can cause serious damage to multiple organs, especially the liver and lungs, which can lead to death if left untreated in time. Based on the type of cysts, strategies for treating CE include surgery, percutaneous management, chemotherapy with benzimidazoles, and, for inactive cysts, a “watch and wait” approach [3]. Over the last few years, the use of pharmacological treatment has increased, suggesting that in the future, drug therapy may replace surgery for uncomplicated cysts [4].

Albendazole (ABZ), a benzimidazole derivative, is the primary anthelminthic drug used to treat this infection. However, its liposoluble nature and poor bioavailability contribute to its efficacy not exceeding 50% in treating CE [3]. Consequently, there is a critical need for the development of new drugs that can overcome these drawbacks and provide improved therapeutic outcomes for patients with this infection.

In this scenario, the use of natural products and their active fractions and components holds great promise as a valuable resource for the development of novel and effective therapies. They exhibit powerful pharmacological activities such as anti‐inflammatory, antioxidant, or antiproliferative effects and have been proven to be accessible, relatively low in cost, and have generally low toxicities [5].

In recent years, there has been a surge in the investigation of various plant extracts as potential therapeutic agents against CE, aiming to identify alternative natural compounds for effective treatment. These studies have yielded numerous encouraging outcomes, highlighting the potential of plant-derived substances in combating CE [6–9].

Hop (*Humulus lupulus* L.) is primarily known as a bittering agent in the brewing industry [10]. However, its uses have diversified in recent times. It is valued in phytotherapy because of its anti-inflammatory, digestive, sedative, and antimicrobial properties [11]. Also the antiparasitic activity was shown for some hoppurified compounds on *Trypanosoma brucei* [12].

The purpose of this study was to assess the *in vitro* efficacy of methanolic extracts from leaves of hop varieties against *E. granulosus sensu stricto* (s.s) protoscoleces.

## 2. Materials and Methods

### 2.1. Plant Material

Hop leaves were collected from a local farm named Granja de Lúpulo MdP, situated nearby Mar del Plata city, Buenos Aires, Argentina (38° 10′ 06″S, 57° 38′ 10″O). To produce methanolic extracts, 100 grams of dry leaves of the hop varieties Bullion, Cascade, Traful, Mapuche, and Victoria were used. They were dried out at room temperature. Voucher specimens (Number: MDQ459, MDQ456, MDQ457, MDQ458, respectively) are available at the Herbarium of Vascular Plants of the Institute of Marine and Coastal Research, National University of Mar del Plata.

### 2.2. Preparation of *H. lupulus* Methanolic Extract

The extraction of secondary metabolites coming from the different varieties was carried out by using the mixture of methanol-water (50 : 50). The extraction was performed by placing 1 gram of leaves (in triplicate) in 30 mL of the solvent for 3 h in an ultrasound bath at 40°C. Then, the suspension was centrifuged for 10 min at 8000 rpm. The obtained supernatant was placed in falcon tubes and conserved at 4°C in dark until use [13].

### 2.3. Chemical Characterization of *H. lupulus* Methanolic Extracts

#### 2.3.1. Quantification of Total Polyphenol Content

The total content of phenolic compounds in the different extracts was determined by the Folin-Ciocalteu method, according to the procedure reported by Singleton and Rossi [14] with some modifications. A calibration curve was made using 0, 2, 6, 8, 10, 15, and 20 *μ*g·ml^−1^ of a standard solution of gallic acid (GA) (200 *μ*g·mL^−1^) and the total polyphenol content of each extract was estimated from the linear regression obtained. The absorbance was measured at 760 nm, and the total polyphenol content was calculated in mg gallic acid equivalents (GAE) per g of dry extract.

#### 2.3.2. Quantification of Total Flavonoid Content

Woisky and Salatino [15] methods with some modifications was used for the determination of total flavonoid content. Briefly, a calibration curve was constructed using 0, 2, 4, 6, 10, 14, 18, and 22 *μ*g·mL^−1^ from a quercetin (QE) standard solution (200 *μ*g·mL^−1^) and the total flavonoid content of each extract was determined from the linear regression obtained. The absorbance was measured at 420 nm using an Agilent 8453 UV–visible spectrophotometer with a diode array, and the total flavonoid content was calculated as mg QE equivalents per g of dry extract.

#### 2.3.3. Quantification of Total Saponin Content

The determination of total saponin content (TSC) was performed according to Le et al. [16], with minor changes. The calibration curve was constructed with oleanoic acid (OA) as a reference standard, and concentrations between 0.001 and 0.005 *μ*g·mL^−1^ of OA were used for the calibration curve. An Agilent 8453 UV-visible spectrophotometer with a diode matrix was used to measure absorbance at 560 nm, and the data was recorded. The TSC of each extract was obtained from the linear regression of the calibration curve and calculated as mg AO equivalent per g of dry extract.

For all the determinations, measurements were carried out in quadruplicates.

### 2.4. Parasite Material and Protoscoleces Collection

Hepatic and pulmonary hydatid cysts were obtained from cattle slaughtered in an abattoir located in the province of Buenos Aires, Argentina. Protoscoleces were removed aseptically from cysts and washed several times with phosphate-buffered saline (PBS, pH 7.2). Viability was assessed by the methylene blue exclusion test [17]. Parasitic material was genotyped by sequencing a fragment of the gene coding for mitochondrial cytochrome c oxidase subunit 1 (CO1), as previously described [18]. The G1 genotype was identified through sequencing analysis.

### 2.5. *In Vitro* Protoscolicidal Activity

Viable and free protoscoleces (2000 per Leighton tube) were cultured in 6 mL of culture medium 199 at 37°C with no change of medium throughout the experiment as previously described [17]. The extracts of the different *H. lupulus* varieties (Bullion, Cascade, Traful, Mapuche, and Victoria) were added at the final concentration of 100 *µ*g/mL. Control protoscoleces were incubated in culture medium or in culture medium with methanol (25 *µ*L/mL). Cultures were performed in triplicate, and each experiment was repeated three times, each time with a different batch of protoscoleces. To determine the appearance of morphological changes, culture tubes were monitored under a microscope daily. Viability assessment using the methylene blue exclusion test was performed every day until day 4 and then, every 3 days. Samples of protoscoleces from each experimental group were periodically taken for ultrastructural studies using scanning electron microscopy (SEM).

### 2.6. Electron Microscopy

Samples of protoscoleces collected in the *in vitro* studies were processed for SEM following the protocol described by Elissondo et al. [19].

### 2.7. Statistical Analysis

All statistical analyses were conducted within the R environment [20]. *P* values less than 0.05 were regarded as statistically significant. For the *in vitro* incubation of protoscoleces with the extracts of the different *H. lupulus* varieties, a generalized linear model (GLM) with a binomial distribution of the error was fitted with the proportion of viability as a response variable and treatments and time in days as explanatory variables. In order to determine if time-treatment interactions should be included in the model, we used the “ANOVA” command from the “car” package [21]. Differences among the *H. lupulus* varieties and control were assessed by pairwise contrasts of the interaction means using the “emmeans” package [22]. Protoscoleces viability is reported as the predicted value of the model with 95% confidence in the upper and lower limits.

### 2.8. Ethic Statement and Experimental Animals

Animal procedures and management protocols were approved by the Institutional Animal Care and Use Committee (RD N° 40/2022) of the Faculty of Exact and Natural Sciences, National University of Mar del Plata, Argentina. The revised form of The Guide for the Care and Use of Laboratory Animals (National Research Council US, 2011) was followed.

The study avoided any unnecessary animal suffering. Animals were housed in a temperature-controlled (22 ± 1°C), light-cycled (12 h light/dark cycle) room and received food and water *ad libitum*.

## 3. Results

Chemical characterization of the different *H. lupulus* varieties is shown in Table 1. The varieties Mapuche and Victoria presented the highest content of flavonoids, total polyphenols, and saponins. The values are showed, as the average of analyses conducted with quadrupled ± standard deviation.

**Figure 1:**
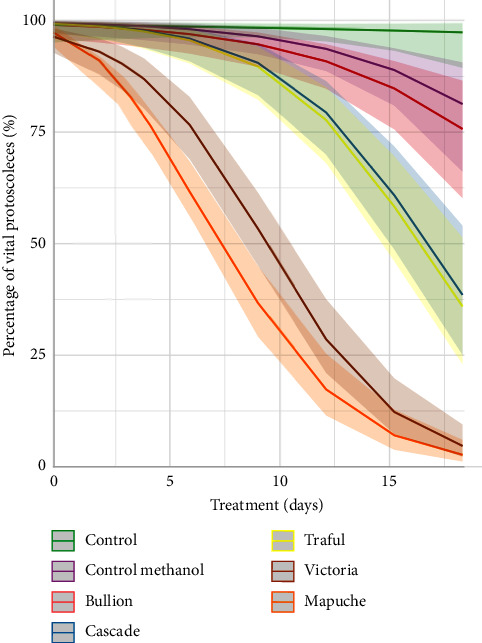
Survival of *E. granulosus* s.s. protoscoleces after *in vitro* exposure to 100 *µ*g/ml of the different *Humulus lupulus* varieties. The ribbons and lines show the 95% confidence intervals and the predicted fits from a generalized linear mixed-effects model.

The survival of *E. granulosus* s.s. protoscoleces incubated with different *H. lupulus* varieties is shown in Figure 1. Varieties Mapuche and Victoria had a stronger effect than varieties Bullion, Cascade, and Traful (*P* < 0.01). The viability of protoscoleces treated with the varieties Mapuche and Victoria decreased to approximately 50% at days 5 y 8, respectively, whereas the treatment with the varieties Traful and Cascade caused a decrease in the viability of 65% at the end of the experiment. The treatment with the variety Bullion did not show any difference with the control (*P*=0.71).

**Figure 2:**
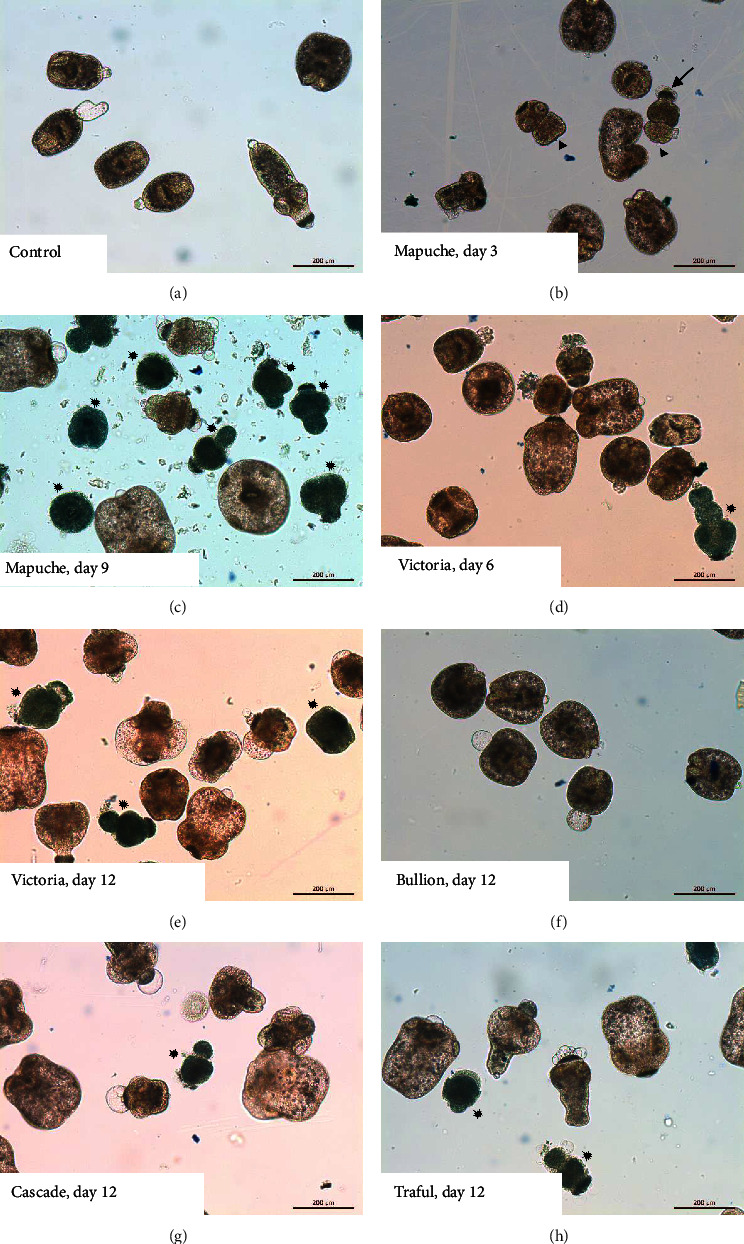
Light microscopy of *E. granulosus* s.s. protoscoleces incubated *in vitro* with the different *Humulus lupulus* varieties. Control protoscoleces remained unaltered throughout the experiment (a). Protoscoleces treated with Mapuche during 3 days showed soma contraction (arrow), rostellar disorganization (arrow head) and presence of blebs in the tegument (b), and after 9 days of treatment an important reduction in vitality was observed (c). The treatment with the variety Victoria caused the first morphological alterations at day 6 (d), which were more pronounced at day 12 (e). The varieties Bullion (f), Cascade (g), and Traful (h) caused a slower effect. Asterisks point dead protoscoleces.

**Figure 3:**
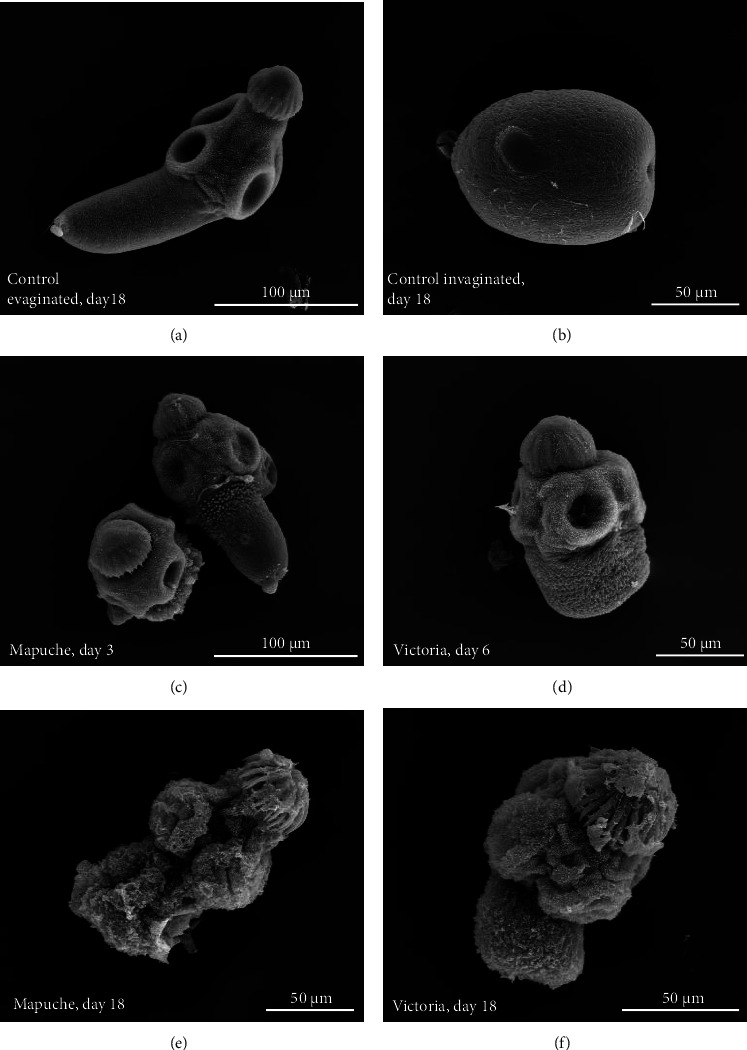
Scanning electron microscopy of *E. granulosus* s.s. protoscoleces incubated *in vitro* with the different *Humulus lupulus* varieties. Control protoscoleces show the characteristic structure: soma (s), suckers (su) and rostellum (r) (a, b). Protoscoleces treated during 3 days with the variety Mapuche (c) and during 6 days with Victoria (d) showed soma contraction and altered microtriches. After 18 days of treatment with both varieties, protoscoleces were completely altered (e, f).

Control protoscoleces cultured in medium 199 or in medium 199 + methanol remained viable (viability >80%) after 18 days of incubation (Figure 1). No alterations in structure were observed throughout the experimental period (Figures 2(a), 3(a), and 3(b)).

The results of the viability test coincided with the tegumental alterations observed daily by an inverted microscope and with the tissue damage determined at the ultrastructural level. Protoscoleces treated with the variety Mapuche showed soma contraction, rostellar disorganization, and presence of blebs in the tegument after 3 days of treatment (Figure 2(b)) and the presence of severe impairment leading to death after 9 days of treatment (Figure 2(d)). The treatment with the variety Victoria caused the first morphological alterations at day 6 (Figure 2(c)) becoming more pronounced at day 12 (Figure 2(e)). The varieties Cascade, Traful, and Bullion caused milder effects (Figures 2(f)–2(h)).

At the ultrastructural level control protoscoleces showed the characteristic structure consisting of the soma region, suckers, and rostellum (Figures 3(a)–3(b)). Protoscoleces treated during 3 days with the variety Mapuche (Figure 3(c)) and during 6 days with the variety Victoria (Figure 3(e)) presented soma contraction and impaired microtriches. After 18 days of treatment with both varieties, protoscoleces were completely altered (Figures 3(d) and 3(f)).

## 4. Discussion

Based on the inadequate therapeutic options available for numerous parasitic infections impacting humans and animals, coupled with the emergence of drug resistance, the utilization of medicinal plants has gained significance as a potential reservoir for the discovery of anti-parasitic drugs [23].

To find new active drugs as potential treatment options for CE, we used *in vitro* cultured *E. granulosus s.s.* protoscoleces to study the activity of different varieties of *H. lupulus* extracts for the first time.

The application of hop as a medicinal plant has more than 2000 years of history [24]. Among their interesting biological properties are their antiviral, antibacterial, antifungal, anti-inflammatory, and anticancerogenic activities. Its strong pharmacological activity and potential therapeutic application are due to the presence of a wide range of bioactive molecules, mainly secondary metabolites which confer particular features to the plant [25].

In the last decades, several phytochemical studies have been conducted to examine the composition of hop cones and other parts of the plant, resulting in the isolation and identification of pharmacologically relevant compounds based on increasing interest in the health benefits of this plant [26, 27].

Over time, breweries have developed distinct varieties of *H. lupulus*, leading to extracts with different properties that may contribute to combating diseases [28].

The secondary metabolites saponins and polyphenols play a crucial role in the plant's defense mechanisms against external biotic and abiotic factors [29].

The composition and amount of secondary metabolites present in hops are mainly dependent on the variety, which is linked to its genetic potential to synthesize certain substances [30].

The varieties Victoria, Mapuche, Cascade, Traful, and Bullion were included in our study. Varieties Mapuche and Victoria showed the highest anthelmintic effect. Interestingly, the variety Mapuche had the highest content of total phenolic compounds (*P* < 0.05), flavonoids (*P* < 0.01), and saponins (*P* < 0.01).

Structural and ultra-structural changes observed by light microscopy and SEM were similar to those reported in previous investigations that studied the anthelmintic effect of medicinal plant extracts [7, 8, 31] or isolated compounds such as carvacrol, thymol, and beta-myrcene against *E. granulosus* s.s protoscoleces [32–34].

In particular, a recent study assessed the hop varieties Victoria and Mapuche against *Varroa destructor*, a parasitic bee mite. Both varieties exhibited significant toxicity against mites; nevertheless, Victoria proved to be the most potent, highlighting its potential as a promising compound for acaricidal treatment [35].

The differences observed in our study between the assessed hop varieties could be attributed to the varying content of secondary metabolites observed in each. It has been stated that saponin compounds, combined with the synergy produced by other components, have a high potential to break cells, hence increasing the cell death process [36]. On the other hand, antimicrobial activity of hop polyphenols was demonstrated. The antimicrobial mechanism involves the interaction with phospholipids of the cell membrane, leading to destabilization, leakage of cellular contents, and ultimately cell death [37].

## 5. Conclusion

The methanolic extracts of the *H. lupulus* varieties Mapuche and Victoria proved to have a significant *in vitro* effect on *E. granulosus* s.s. protoscoleces. The beer-making industry exclusively uses hop cones, leaving behind large amounts of hop leaves as an agricultural by-product that is not being utilized. On the basis of our study, we propose that hop leaves could also be used as a source of secondary metabolites with anthelmintic activity.

## Figures and Tables

**Table 1:** Content of total phenolic compounds, total flavonoids and total saponins of the different *Humulus lupulus* varieties extracts.

Varieties	Total phenolic compound *µ*g AG mL^−1^ extract	Total flavonoids *µ*g Q mL^−1^ extract	Total saponins *µ*g AO mL^−1^ extract
Mapuche	74 ± 4	62 ± 6	126 ± 6
Victoria	62 ± 3	52 ± 5	83 ± 4
Bullion	35 ± 2	26 ± 2	33 ± 2
Cascade	34 ± 2	30 ± 2	34 ± 2
Traful	32 ± 2	28 ± 1	29 ± 1

AG: gallic acid; Q: quercetine; AO: oleanoic acid.

## Data Availability

The data used to support the findings of this study are available from the corresponding author upon request.

## Additional Points

*Key Findings*. The use of natural products provides an interesting tool for the discovery of new relevant treatments for cystic echinococcosis. The methanolic extracts of leaves of the *Humulus lupulus* varieties Mapuche and Victoria demonstrated a marked *in vitro* effect against *Echinococcus granulosus* s.s. protoscoleces. The treatment of *Echinococcus granulosus* s.s. protoscoleces with the *Humulus lupulus* varieties Mapuche and Victoria caused a severe impairment both at the structural and ultrastructural level.
